# Photoprotective and immunoregulatory capacity of ginsenoside Rg1 in chronic ultraviolet B-irradiated BALB/c mouse skin

**DOI:** 10.3892/etm.2013.1235

**Published:** 2013-07-25

**Authors:** JIN-SHU LOU, XIAO-E CHEN, YAN ZHANG, ZUO-WEN GAO, TAI-PING CHEN, GUO-QIANG ZHANG, CHANG JI

**Affiliations:** 1 Department of Medical Oncology, The 86th Hospital of the Dangtu, Dangtu, Anhui 243100;; 2 Department of Dermatology and Venereology, Nanjing Jingdu Hospital, Nanjing, Jiangsu 210002, P.R. China

**Keywords:** ginsenoside Rg1, UVB irradiation, p53 protein, cytokine, BALB/c mouse skin

## Abstract

The aim of this study was to investigate the photoprotective and immunoregulatory capacities of ginsenoside Rg1 in skin irradiated by chronic ultraviolet B (UVB) and to verify the potential mechanisms of action. BALB/c mice were pretreated with a topical application of ginsenoside Rg1 and irradiated with different doses of UVB daily for 30 consecutive days. Following chronic UVB irradiation, there were significant pathological changes in the skin of the BALB/c mice, including hyperkeratosis, acanthosis, sponge-like edematization and sunburn occurring in the epidermis, while edema, telangiectasis and inflammatory cell infiltration were observed in the papillary layer of the dermis. Treatment with ginsenoside Rg1 was able to reduce such changes induced by UVB irradiation. The number of p53 protein-positive stained cells following UVB irradiation was also observed by immunohistochemical analysis. Ginsenoside Rg1 downregulated the p53 protein expression induced by UVB irradiation, leading to reductions of 69.50, 23.53 and 12.93% at doses of 30, 60 and 120 mJ/cm^2^, respectively. Using reverse transcription polymerase chain reaction (RT-PCR), reductions in the levels of interferon (IFN)-γ mRNA expression were detected following UVB exposure; reductions of 19.6, 36.3 and 39.6% were observed following UVB irradiation at doses of 30, 60 and 120 mJ/cm^2^, respectively. The interleukin (IL)-10 mRNA expression levels increased by 40.1, 71.0 and 89.4% and the tumor necrosis factor (TNF)-α mRNA expression levels increased by 36.4, 18.4 and 8.6% following UVB irradiation at doses of 30, 60 and 120 mJ/cm^2^, respectively. However, pretreatment with ginsenoside Rg1 was observed to markedly attenuate the UVB irradiation-induced effects on the mRNA expression levels of the three cytokines. The topical application of ginsenoside Rg1 was able to protect the irradiated skin from UVB injury and reduce UVB-induced p53 protein expression. Ginsenoside Rg1 also demonstrated a potential regulatory effect on the UVB-induced local expression of the mRNA of the cytokines IFN-γ, IL-10 and TNF-α, which may be important in its immunoregulatory and inflammatory mechanisms.

## Introduction

Ultraviolet (UV) irradiation, particularly UVB, has suppressive effects on the immune system. UVB-induced immunosuppression and photodamage are risk factors for skin cancer development in animals and humans (1–3). The activation of the p53 gene is important for photodamage/repair, cell cycle arrest, cellular apoptosis and photocarcinogenesis (4). It has been demonstrated that numerous cytokines are involved in the process of UVB-induced inflammation and/or immunosuppression (5,6). With regard to the T-helper (Th)1/Th2 balance, Th1 cells secrete interferon (IFN)-γ and IL-2, and Th2 cells secrete IL-4, IL-5 and IL-10. Certain potential mechanisms for UVB-induced photodamage and immunosuppression involve the expression and activation of the p53 protein, as well as an alteration in the balance of Th1- and Th2-associated cytokines (7,8).

There has been a particular focus on the chemoprevention of photodamage, which is considered as a less toxic and more effective approach than traditional methods. Ginsenoside is extracted from the root, stem and leaves of ginseng, and consists of three major moieties: Rg, Rb and Rh (9). Ginsenoside Rg1 is a member of the protopanaxatriol group of compounds, which exhibit multiple pharmacological effects (10). A recent study observed that ginsenoside Rg1 attenuated the UVB-induced G1 arrest in HaCaT cells and dermal fibroblasts through downregulating the expression of p16, p21 and p53 (11). Consistent with other studies, our previous studies have shown that 8-methoxypsoralen (8-MOP)/UVA-irradiated fibroblasts pretreated with ginsenoside Rg1 demonstrated a reduction in the expression of senescence-associated β-galactosidase (SA-β-gal), a down-regulation in the level of senescence-associated proteins and a deceleration in telomere shortening (12). These results suggest that ginsenoside Rg1 significantly antagonizes premature senescence induced by 8-MOP/UVA in fibroblasts. However, it has not yet been elucidated whether the photoprotective and immunoregulatory mechanisms of ginsenoside Rg1 are correlated with these cytokines in BALB/c mice. This study focused on the photoprotective capacity of ginsenoside Rg1 and its immunoregulatory capacity on the changes in IFN-γ, IL-10 and TNF-α cytokines induced by chronic UVB irradiation in BALB/c mouse skin.

## Materials and methods

### Experimental animals

Female BALB/c mice, aged 6–8 weeks old and weighing ∼20–25 g, were obtained from the Chinese Academy of Science, Shanghai SLAC Laboratory Animal Co., Ltd. (Shanghai, China). A total of six mice were used in each group. Mice were housed in a pathogen-free barrier facility in the Experimental Animal Center of Nanjing Medical University (Nanjing, China) and all experiment protocols were approved by The Animal Care and Use Committee of Nanjing Medical University.

### Reagents

Purified ginsenoside Rg1 was purchased from Sigma (St. Louis, MO, USA) and the ginsenoside Rg1 solution was prepared as follows: 3 mg ginsenoside Rg1 was dissolved in 100 *μ*l acetone (3.0 mg, 6.5 *μ*mol ginsenoside Rg1 in 100 *μ*l acetone/3 cm^2^ mouse skin). Polyclonal antibodies to mouse p53 (sc-6243) were purchased from Santa Cruz Biotechnology, Inc. (Santa Cruz, CA, USA).

### UVB irradiation protocols

UVB (280–320 nm) radiation was used, according to the experiment. The UVB source (Spectronics Corp., Lincoln, NE, USA) emitted an average irradiation of 1,243 mW/cm^2^. BALB/c mice were randomly divided into groups as follows: (i) sham-irradiated group, mice subjected to a sham UVB irradiation procedure; (ii) UVB irradiation only; (iii) ginsenoside Rg1 pretreatment plus UVB exposure; and (iv) acetone pretreatment plus UVB exposure, as the control. Prior to UVB exposure, all mice were shaved with electric clippers and were treated topically with ginsenoside Rg1 solution, acetone or nothing for 30 min, and then certain dosages of UVB (30, 60 and 120 mJ/cm^2^) were delivered at each exposure, respectively, for 30 consecutive days.

### Skin tissue treatment

At the end of experiment, the treated skin of the ear and dorsal areas were obtained. For histopathological examination and immunohistochemical analysis, the ear biopsies were placed in 10% phosphate-buffered formalin, then dehydrated in ascending concentrations of ethanol, cleared in xylene and embedded in paraffin. Following conventional treatment, 4 mm-thick tissue sections were prepared for regular hematoxylin and eosin staining or immunohisto-chemical staining. For reverse transcription polymerase chain reaction (RT-PCR) detection, the dorsal tissue was preserved in freezing conditions (−70°C). Total RNA was isolated using a total RNA extraction kit (TRIzol^®^ reagent; Molecular Research Centre, Inc., Cincinnati, OH, USA). The quality of the RNA was confirmed by measuring the optical density (OD) 260/280 ratio.

### Immunohistochemical analysis of p53 protein expression

The sections were treated with 0.01 M sodium citrate buffer, prior to incubation with primary antibodies against p53 (Santa Cruz Biotechnology, Inc.) and incubation in a moist chamber overnight at 4°C. After being washed in phosphate-buffered saline (PBS), the sections were incubated in secondary antibody, immunoglobulin (Ig)G, for 20 min and then were incubated with the streptavidin-biotin-peroxidase complex (SABC) and 3,3′-diaminobenzidine (DAB) substrate solution, respectively. Subsequently, the slides were counterstained with hematoxylin and observed under a light microscope. A positive result was shown as a light to dark brown staining/precipitate in the nuclei of the cells. The p53^+^ nuclei in the epidermis among each 200 basal cells were counted in 10 randomly selected visual fields at ×400 magnification.

### RT-PCR measurement

The mRNA expression levels of the IFN-γ, IL-10 and TNF-α genes were detected by RT-PCR. In brief, cDNAs were synthesized from 1 *μ*l total RNA using avian myeloblastosis virus (AMV) reverse transcriptase (Takara Co., Ltd., Shiga, Japan) and random oligo (dT) primers. PCR amplification was performed with a thermal cycler (Geneamp^®^ PCR System 2400, Perkin-Elmer, Norwalk, CT, USA). Thirty-five PCR cycles were run under the following conditions: DNA denaturation at 94°C for 1 min; primer annealing at 60°C for IFN-γ and 56°C for IL-10 for 1 min and at 57°C for TNF-α for 40 sec; and DNA extension at 72°C for 1 min. The housekeeping gene β-actin was amplified as an internal control from the same cDNA product in a separate reaction. The primer sequences (Shanghai BioAsia Co. Ltd, Shanghai, China) specific for the previously mentioned cytokines (IFN-γ, IL-10, TNF-α and β-actin) were as follows: IFN-γ gene (426 bp), 5′-GGCTGTTTCTGGCTG TTACTGC-3′ (upstream) and 5′-GACTCCTTTTCCGCT TCCTGA-3′ (downstream); IL-10 gene (394 bp), 5′-CAA TAACTCACCCACTTCC-3′ (upstream) and 5′-CAT GGCCTTGTAGACACCTT-3′ (downstream); TNF-α gene (212 bp), 5′-TCTCATCAGTTCTATGGCCC-3′ (upstream) and 5′-GGGAGTAGACAAGGTACAAC-3′ (downstream); and β-actin gene (222 bp), 5′-TGACCGGCTTGTATGCTATC-3′ (upstream) and 5′-CAGTGTGAGCCAGGATATAG-3′ (downstream).

All the PCR products were subjected to electrophoresis in a 2.0% agarose gel containing 0.5 mmol/l ethidium bromide and photographic images were subsequently captured. The density of each band was analyzed using a gel imaging system densitometer (Bio-Rad, Hercules, CA, USA).

### Statistical analysis

The results are expressed as the mean ± standard deviation. The statistical significance of the difference between two independent groups was determined using a t-test. P<0.05 was considered to indicate a statistically significant difference.

## Results

### Ginsenoside Rg1 protects against photodamage caused by UVB irradiation in the skin of BALB/c mice

**Figure 1.**
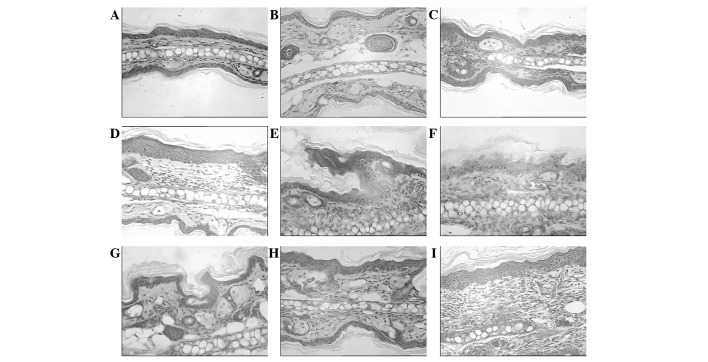
Histopathology of hematoxylin and eosin-stained specimens in different groups of BALB/c mice (magnification, ×400): (A) control mice; (B) mice pretreated with ginsenoside Rg1 only; (C) mice pretreated with acetone plus ultraviolet B (UVB) irradiation; (D–F) mice irradiated with different doses of UVB (30, 60 and 120 mJ/cm^2^, respectively); (G–I) mice pretreated with ginsenoside Rg1 plus UVB irradiation (30, 60 and 120 mJ/cm^2^, respectively).

The histological changes in the irradiated and non-irradiated control mouse skin samples were examined 30 days subsequent to the UVB-irradiation at different doses. Fig. 1 demonstrates the pathological results of the hematoxylin and eosin-stained specimens observed in the different groups of BALB/c mice. The UVB-exposed mouse skin appeared thicker compared with that of the mice in the control and ginsenoside Rg1-pretreated groups, with hyperkeratosis, acanthosis, sponge-like edematization and sunburn occurring in the epidermis. In addition, edema in the papillary layer of the dermis, telangiectasis, an intense diffuse inflammatory leukocyte infiltration (predominantly monocytes/macrophages and neutrophils) and tissue necrosis were observed in the UVB-irradiated mice. Following chronic UVB irradiation, infiltrating cells were present in the dermis in higher numbers, compared with those in the non-UVB-exposed skin of the control mice. The damage was more marked in the mice exposed to a higher dosage of UVB irradiation.

The application of ginsenoside Rg1 protected against UVB-induced damage and maintained the normal structure of the epidermis and dermis. A significant alleviation of skin swelling, slight epidermal injury and a reduced lymphocyte and polymorphonuclear cell infiltration with minimal dermal tissue destruction were observed in the groups pretreated with ginsenoside Rg1 compared with that in the UVB-irradiated mice that did not receive pretreatment. Therefore, morphological differences between the irradiation-only mouse skin samples and samples pretreated with ginsenoside Rg1 were able to be distinguished in the histopathological images.

In addition, to examine whether ginsenoside Rg1 exerted a toxic effect on the skin of BALB/c mice, one group was treated with ginsenoside Rg1 without UVB irradiation. The result demonstrated that the morphology of sham-irradiated skin treated with ginsenoside Rg1 for 1 month appeared similar to the skin of the normal control mice. Thus, it was concluded that 30 mg/ml ginsenoside Rg1 did not affect the histopathology of the mouse skin. For the group treated with acetone plus UVB irradiation, the pathological changes appeared to be similar to those in the UVB-irradiated mouse skin, which suggested that the acetone solvent possessed no marked photoprotective capacity on the UVB-irradiated mouse skin.

### Ginsenoside Rg1 downregulates p53 protein expression induced by UVB irradiation in the skin of BALB/c mice

**Figure 2.**
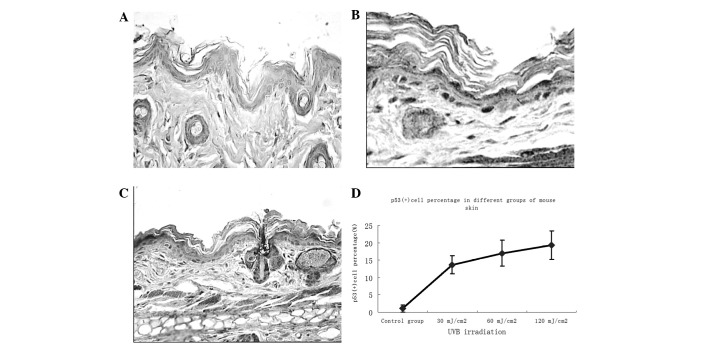
Effect of chronic ultraviolet B (UVB) irradiation on the p53 protein expression in mouse epidermis. The skin biopsies in BALB/c mice were performed 30 days after consecutive specific treatments, and immunohistochemical analysis was used to determine the number of p53-positive stained cells. The immunohistochemical analysis is shown in (A–C). Compared with the control group (A), the majority of the p53^+^ cells appeared in the basal layer of the epidermis (B), while some were present around the hair follicles (C). (D) Effect of different doses of UVB on p53 protein expression in the skin of BALB/c mice. The combined data of three representative experiments are shown and expressed as the mean percentage value ± standard deviation.

**Figure 3.**
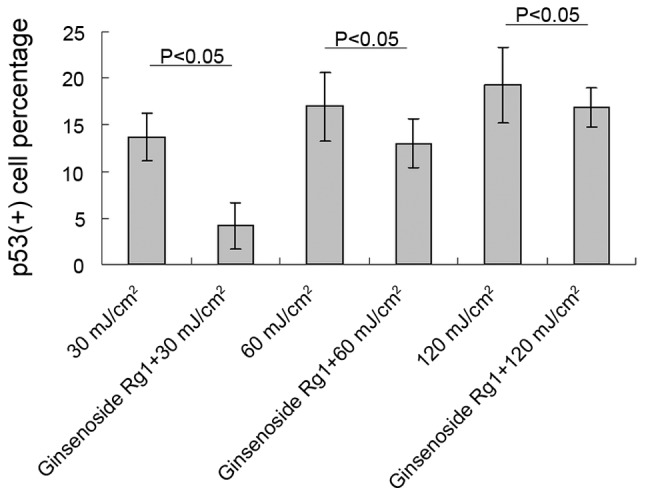
Downregulation of p53 protein expression by ginsenoside Rg1 in ultraviolet B (UVB)-induced p53-positive cells in mouse skin. The skin of BALB/c mice was pretreated with 3.0 mg/100 *μ*l ginsenoside Rg1 and then irradiated with 30, 60 and 120 mJ/cm^2^ UVB irradiation. A significant difference was observed between the ginsenoside Rg1-pretreated and UVB irradiation-only groups at each UVB dose. Ginsenoside Rg1 was able to reduce the p53 protein expression by 69.50, 23.53 and 12.93% at 30, 60 and 120 mJ/cm^2^ UVB irradiation, respectively. The combined data of three representative experiments are shown and expressed as the mean percentage value ± standard deviation.

To examine the effects of different doses of UVB irradiation and ginsenoside Rg1 on local p53 expression in BALB/c mice, skin biopsies were performed following 30 days of consecutive specific treatments and immunohistochemical analysis was used to determine the number of p53^+^ stained cells (which were stained brown). Subsequent to UVB exposure, the majority of the p53^+^ cells appeared in the basal layer of the epidermis, while some were present around the hair follicles in the skin of irradiated mice (Fig. 2). The number of p53^+^ cells was markedly increased in comparison with that in the non-UVB-exposed skin, with a noticeable upregulation at 30 mJ/cm^2^ and marginal increases at 60 and 120 mJ/cm^2^. Abundant p53^+^ cells were observed in the skin of the UVB-irradiated groups; however, only a few p53^+^ cells remained in the group pretreated with ginsenoside Rg1. Significant differences between the ginsenoside Rg1-pretreated and the UVB irradiation-only groups were observed at each parallel control under 30, 60 and 120 mJ/cm^2^ UVB irradiation (Fig. 3). With regard to the p53 protein expression induced by UVB irradiation, pretreatment with ginsenoside Rg1 resulted in marked reductions of 69.50, 23.53 and 12.93% at 30, 60 and 120 mJ/cm^2^, respectively. However, in the acetone-pretreated mice, the number of p53^+^ nuclei was 78% of the number in the UVB irradiation group (data not shown), which further indicated that acetone did not have any photoprotective capacity under such circumstances.

### Different doses of multiple UVB irradiation modulate the mRNA expression of three types of cytokines, leading to the downregulation of IFN-γ and upregulation of IL-10 and TNF-α

**Figure 4.**
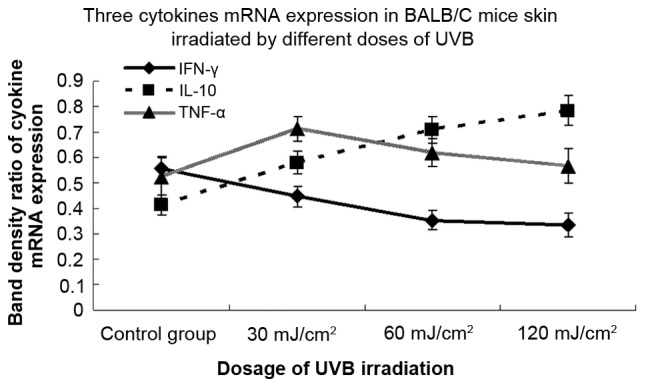
Effects of different doses of ultraviolet B (UVB) irradiation on the mRNA expression levels of three types of cytokines. BALB/c mice were irradiated with different doses of UVB irradiation (30, 60 and 120 mJ/cm^2^) for 30 consecutive days, skin samples were collected and the mRNA expression of interferon (IFN)-γ, interleukin (IL)-10 and tumor necrosis factor (TNF)-α genes was detected by a reverse transcription polymerase chain reaction (RT-PCR). The mRNA expression of the IFN-γ cytokine was downregulated by 19.6, 36.3 and 39.6% following 30, 60 and 120 mJ/cm^2^ UVB irradiation, respectively (P<0.05). The levels of IL-10 mRNA in the UVB-irradiated group were upregulated in a dose-dependent manner, showing 40.1, 71.0 and 89.4% increases following 30, 60 and 120 mJ/cm^2^ UVB irradiation, respectively (P<0.05). An upregulation of TNF-α mRNA was observed in the UVB-irradiated groups by 36.4% at 30 mJ/cm^2^ (P<0.05) and 18.4 and 8.6% at 60 and 120 mJ/cm^2^ (P>0.05), respectively, compared with the control group. The combined data of three representative experiments are shown and expressed as the mean band density value ± standard deviation.

To examine the effects of different doses of multiple UVB irradiation on the local expression of the IFN-γ, IL-10 and TNF-α genes, skin samples were collected following 30 consecutive days of irradiation and processed for mRNA detection by RT-PCR. There were statistically significant differences in the mRNA expression of cytokines between mice irradiated with different doses of UVB and non-irradiated control mice (Fig. 4). Compared with the sham-irradiated group, the mRNA expression of the IFN-γ cytokine was downregulated by 19.6, 36.3 and 39.6% following 30, 60 and 120 mJ/cm^2^ UVB irradiation, respectively, and the difference was statistically significant. In addition, the levels of IL-10 mRNA in the irradiated groups demonstrated a dose-dependent induction and increased by 40.1, 71.0 and 89.4% following 30, 60 and 120 mJ/cm^2^ UVB irradiation, respectively, and the upregulation was statistically significant at each dose of UVB. Furthermore, the expression of TNF-α mRNA in each UVB-irradiated group was upregulated by 36.4, 18.4 and 8.6% versus the sham-irradiated group, respectively. The expression level of TNF-α mRNA peaked significantly at 30 mJ/cm^2^ and fell marginally at 60 and 120 mJ/cm^2^.

### Pretreatment with ginsenoside Rg1 prior to UVB exposure may reverse UVB-induced mRNA expression levels of IFN-γ, IL-10 and TNF-α

**Figure 5.**
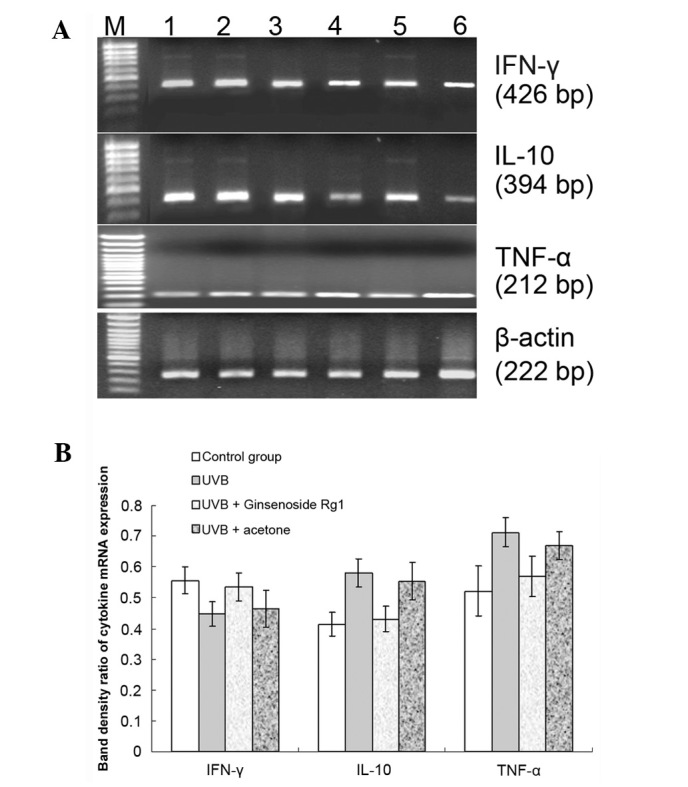
Effect of ginsenoside Rg1 pretreatment on the ultraviolet B (UVB)-induced mRNA expression levels of three cytokines. BALB/c mice were pretreated with 3.0 mg/100 *μ*l ginsenoside Rg1 and then irradiated with 30 mJ/cm^2^ UVB for 30 consecutive days. A reverse transcription polymerase chain reaction (RT-PCR) was used to detect the mRNA expression of inter-feron (IFN)-γ, interleukin (IL)-10 and tumor necrosis factor (TNF)-α genes for the conditioned skin samples. (A) RT-PCR results for IFN-γ, IL-10 and TNF-α mRNA expression on agarose gel electrophoretograms from the different conditioned skin samples of BALB/c mice: Lane M, DNA marker; lane 1, 120 mJ/cm^2^ UVB irradiation; lane 2, 60 mJ/cm^2^ UVB irradiation; lane 3, acetone plus 30 mJ/cm^2^ UVB irradiation; lane 4, ginsenoside Rg1 plus 30 mJ/cm^2^ UVB irradiation; lane 5, 30 mJ/cm^2^ UVB irradiation; lane 6, sham-irradiated group. (B) Ginsenoside Rg1 increased the mRNA level of IFN-γ by 19.7% and inhibited the UVB-induced IL-10 and TNF-α mRNA expression by 25.7 and 20%, respectively. However, acetone had no significant effect on the mRNA expression of the three cytokines (P>0.05). The combined data of three representative experiments are shown and expressed as the mean band density value ± standard deviation.

While UVB exposure reduced IFN-γ mRNA expression and induced IL-10 and TNF-α mRNA expression in the skin of BALB/c mice (Fig. 4), pretreatment with ginsenoside Rg1 prior to UVB exposure resulted in an attenuation of the effects on the expression of the cytokines caused by 30 mJ/cm^2^ UVB irradiation (Fig. 5). The conditioned skin samples were collected and the RT-PCR results revealed that ginsenoside Rg1 increased the UVB-reduced IFN-γ mRNA level by 19.7% and led to significant reductions in the mRNA expression levels of IL-10 and TNF-α of 25.7% and 20%, respectively. Unlike ginsenoside Rg1, acetone had no such effect on the mRNA expression of the three cytokines.

## Discussion

UVB irradiation is the major environmental carcinogen for human skin. The biologically active UVB wavelengths are predominantly absorbed in the epidermis, where UVB directly damages DNA via formation of mutagenic photoproducts (13). It has been demonstrated that leukocyte infiltration into UVB-irradiated skin is critical in UVB-induced inflammation and the immunological response (14). In order to understand the mechanism by which ginsenoside Rg1 affects p53 protein expression and certain cytokine changes induced by UVB irradiation, the present study was conducted on BALB/c mice by pretreating the mice with ginsenoside Rg1 prior to 30 days of UVB exposure. The histopathological results suggested that ginsenoside Rg1 protected against UVB-induced photodamage to the skin, and resulted in an alleviation of skin swelling, a relief of epidermal injury and dermal destruction and a reduction in the inflammatory cell infiltration. Of particular importance was the fact that the number of p53^+^ cells in the UVB-exposed epidermis was higher than that in the control, showing a noticeable upregulation at a dose of 30 mJ/cm^2^ and marginal increases at doses of 60 and 120 mJ/cm^2^. This type of curved pattern indicated that particularly high doses of UVB irradiation injured the epidermis, leading to cell death and broken cells, with the result that p53^+^ cells were not able to be stained and detected. This further suggested that UVB irradiation damaged the epidermal cells and upregulated p53 protein expression in intact cells. Pretreatment with ginsenoside Rg1 significantly reduced the number of p53^+^ cells in the epidermis, particularly in the low dose UVB-irradiated group. Ginsenoside Rg1 treatment resulted in a marked reduction in p53 protein expression by 69.50% at a dose of 30 mJ/cm^2^ and smaller reductions for the groups irradiated at doses of 60 and 120 mJ/cm^2^, which may indicate that the protection of ginsenoside Rg1 is limited, since high doses of UVB irradiation destroy epidermal cells. These data suggest that a possible mechanism of the photoprotective capacity of ginsenoside Rg1 may be associated with the downregulation of p53 protein expression.

UVB irradiation impairs the cell-mediated immune response by means of reducing the levels of Th1 cytokines (such as IFN-γ, IL-2 and IL-12) and inducing Th2 cytokines (such as IL-10 and IL-4). An imbalance of the Th1 versus Th2 cell cytokines may be responsible for the development of UVB-induced immunosuppression (15). The aim of the study was to clarify in an *in vivo* experiment whether UVB-induced immunosuppression was associated with the downregulation of IFN-γ and/or the upregulation of IL-10 and TNF-α, and whether the topical application of ginsenoside Rg1 prior to UVB irradiation was able to reverse the immunosuppressive changes caused by UVB irradiation. The study demonstrated that UVB exposure led to a reduction in IFN-γ mRNA expression levels and increases in IL-10 and TNF-α mRNA expression levels and that ginsenoside Rg1 was able to attenuate these phenomena to a certain degree, i.e. by upregulating IFN-γ and/or downregulating IL-10 and TNF-α in the skin of BALB/c mice.

With regard to the three types of cytokines, IFN-γ and IL-10 represent Th1/Th2 development and perform different functions to maintain the balance of Th1/Th2 immunity (16,17). TNF-α is not only associated with immuno-suppression, but also acts as an inflammatory mediator (18,19). IFN-γ, serving as one type of Th1 cytokine, is able to stimulate antigen presenting cells (APCs) and promote the cell-mediated immune response (20). IL-10, which is identified as a Th2 cell product, is an important regulator of cutaneous immune function and has been demonstrated to be involved in UVB-induced immunosuppression (15,21,22). In addition, IL-10 inhibits Th1 cell clones by downregulating IFN-γ expression and it also reduces antigen presentation by APCs, including epidermal Langerhans cells (23). With regard to the immunity balance in the skin, IFN-γ and IL-10, are involved in modulating the immunity of the skin. It has been demonstrated that numerous factors are able to stimulate TNF-α production, and UVB is one of the major stimuli for TNF-α production in the surrounding environment (24,25). In addition, the results of the present study indicated that TNF-α and IL-10 signaling was involved in UVB-induced immunosuppression, and was important in the UVB-induced immunosuppressive mechanism.

An additional aim of the study was to explore whether ginsenoside Rg1 application at the site of UVB irradiation was capable of repairing the impaired immune response. On the basis of previous results, it was speculated that the immunoprotective effect of ginsenoside Rg1 resulted from the enhancement of host immunity through the upregulation of Th1 cytokines and the prevention of the UVB-induced cytokines from decreasing. In the present animal model, ginsenoside Rg1 application prior to UVB exposure exerted an effect on the immune response in favor of Th1 cytokine production. Our data showed that it was likely that the immunoprotective capacity of ginsenoside Rg1 was mediated by the upregulation of IFN-γ and the downregulation of IL-10 mRNA expression, thereby acting against the effects induced by UVB irradiation. In addition, ginsenoside Rg1 inhibited UVB-induced TNF-α mRNA expression in BALB/c mice, which led to protection against UVB-induced inflammation. It was concluded that the anti-inflammatory effect of ginsenoside Rg1 was mediated in part through the downregulation of TNF-α mRNA expression. In combination, the data strongly indicated that ginsenoside Rg1 may be a potential immunomodulator and anti-inflammatory substance against UVB-induced immuno-suppression and inflammation. The study demonstrated that the local application of ginsenoside Rg1 may provide a novel method for cutaneous photoprotection and the treatment of immune-mediated skin diseases. The data also showed that it was ginsenoside Rg1 itself, rather than acetone, that provided the photoprotective effects against UVB-induced photo-damage and photoimmunological alteration, since there was no significant difference between the acetone pretreatment and UVB groups. This implied that unlike ginsenoside Rg1, acetone did not demonstrate any photoprotective capacity.

In conclusion, the present study demonstrated that chronic UVB irradiation induced dose-dependent histopathological changes and affected the levels of p53 protein expression in the skin of BALB/c mice, and that pretreatment with topical ginsenoside Rg1 was able to protect the mouse skin from photodamage and p53 expression. In addition, pretreatment with ginsenoside Rg1 resulted in an attenuation of the up- or downregulation of the mRNA expression of three cytokines (IFN-γ, IL-10 and TNF-α) induced by UVB exposure, suggesting a potential mechanism by which ginsenoside Rg1 prevents UVB-induced local immunosuppression. The results suggest that ginseng may be a potential photocarcinogenesis inhibitor for humans.
